# Developmental and Repairing Production of Myelin: The Role of Hedgehog Signaling

**DOI:** 10.3389/fncel.2018.00305

**Published:** 2018-09-06

**Authors:** Yousra Laouarem, Elisabeth Traiffort

**Affiliations:** Small Molecules of Neuroprotection, Neuroregeneration and Remyelination – U1195, INSERM, University Paris-Sud/Paris-Saclay, Kremlin-Bicêtre, France

**Keywords:** oligodendrocyte, brain, spinal cord, smoothened, Gli

## Abstract

Since the discovery of its role as a morphogen directing ventral patterning of the spinal cord, the secreted protein Sonic Hedgehog (Shh) has been implicated in a wide array of events contributing to the development, maintenance and repair of the central nervous system (CNS). One of these events is the generation of oligodendrocytes, the glial cells of the CNS responsible for axon myelination. In embryo, the first oligodendroglial cells arise from the ventral ventricular zone in the developing brain and spinal cord where Shh induces the basic helix-loop-helix transcription factors Olig1 and Olig2 both necessary and sufficient for oligodendrocyte production. Later on, Shh signaling participates in the production of oligodendroglial cells in the dorsal ventricular-subventricular zone in the postnatal forebrain. Finally, the modulation of Hedgehog signaling activity promotes the repair of demyelinated lesions. This mini-review article focuses on the Shh-dependent molecular mechanisms involved in the spatial and temporal control of oligodendrocyte lineage appearance. The apparent intricacy of the roles of two essential components of Shh signaling, Smoothened and Gli1, in the postnatal production of myelin and its regeneration following a demyelinating event is also highlighted. A deeper understanding of the implication of each of the components that regulate oligodendrogenesis and myelination should beneficially influence the therapeutic strategies in the field of myelin diseases.

## Introduction

The generation of oligodendrocyte progenitor cells (OPCs) comprises several spatiotemporal waves localized firstly in ventral regions of the central nervous system (CNS) and slightly later in more dorsal domains. Although most generated OPCs differentiate into mature oligodendrocytes leading to CNS myelination, a small fraction remains in a slowly proliferative or quiescent state in the adult tissue (Dawson et al., 2003). After a demyelinating insult, these OPCs constitute one of the cell populations responsible for myelin repair (Franklin et al., 1997). Dorsal and ventral OPCs display intrinsic differences regarding migration and differentiation capacities when they are considered in a similar environment. These differences are more apparent in progenitors derived from the adult brain than from the neonatal brain (Crawford et al., 2016). Besides OPCs, neural stem cells(NSCs) or neuroblasts located in the ventricular-subventricular zone (V-SVZ) can also generate oligodendroglial cells after demyelination (Picard-Riera et al., 2002; Cayre et al., 2006; Menn et al., 2006; Aguirre et al., 2007). However, OPCs and NSCs differently contribute to remyelination according to the demyelination model that is used or the brain region that is considered (Xing et al., 2014; Brousse et al., 2016; Kazanis et al., 2017).

**FIGURE 1:**
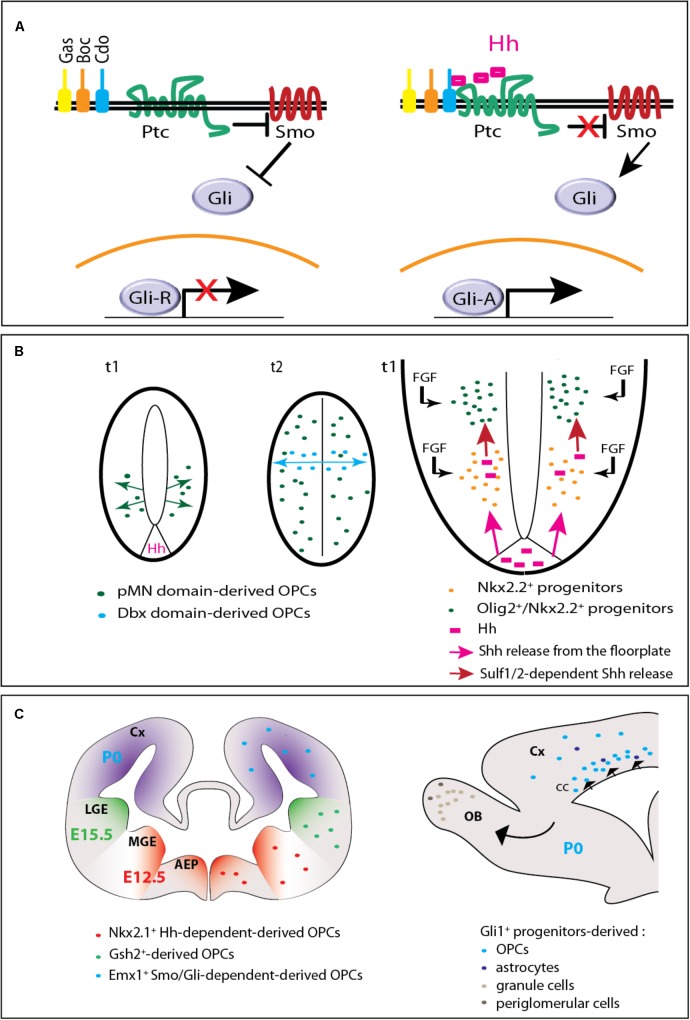
Hedgehog signaling in embryonic and postnatal oligodendrogenesis. **(A)** The Hedgehog signaling pathway. In the absence of Hedgehog (left), the receptor Patched (Ptc) exerts a repressive activity on Smoothened (Smo). When Hedgehog (Hh) binds Ptc and one of its co-receptors (Gas, Boc, or Cdo), Smo repression is relieved triggering an intracellular signaling cascade that involves the transcription factors of the Gli family. The Gli activator (GliA) instead of the Gli repressor (GliR) form is then transported toward the nucleus and subsequently leads to the transcription of target genes. **(B)** Hedgehog-dependent OPC production in the embryonic spinal cord. On the left (adapted from Fogarty et al., 2005), two cross-sectional views of the neural tube are shown. t1 and t2 indicate the embryonic days when ventral and dorsal OPCs are produced, respectively. At t1, OPCs arise from the pMN domain in a Hedgehog (Hh)-dependent manner. At t2, while pMN-derived OPCs have invaded the whole neural tube, a second wave arises from the Dbx domain in a Hedgehog-independent manner. On the right, a magnified scheme of the ventral part of the neural tube shows that Hedgehog (pink) is secreted from both the floorplate (the ventral most part of the neural tube) and Nkx2.2^+^ cells (just above) that upregulate the expression of the morphogen when they become exposed to fibroblast growth factors (FGFs). Because they are submitted to higher Hedgehog signaling activity and to FGF, adjacent Olig2^+^ progenitors give rise to OPCs. **(C)** Hedgehog-dependent OPC production in the forebrain. On the left, the scheme (adapted from Kessaris et al., 2006) represents a coronal view of the mouse forebrain and the three successive waves of OPC production at the embryonic (E12.5, E15.5) and early postnatal (P0) periods. AEP, anterior entopeduncular area; Cx, cerebral cortex; MGE, medial ganglionic eminence; LGE, lateral ganglionic eminence. On the right, a sagittal view of the forebrain indicates the progeny of Gli1^+^ neural progenitors derived from the dorsal V-SVZ at P0. The progeny comprises OPCs and astrocytes migrating to the corpus callosum (cc) and cerebral cortex (Cx), granule and periglomerular neurons migrating to the olfactory bulbs (OB).

The signaling pathway induced by the secreted proteins Hedgehog has been discovered three decades ago and is well-known as a regulator of oligodendrocyte and myelin production. The transduction of the Hedgehog signal classically involves a major receptor complex associating the 12-pass transmembrane protein Patched (Ptc) and one of its co-receptors including Cell adhesion molecule-related, down-regulated by oncogenes (Cdo), Brother of Cdo (Boc), or Growth-arrest-specific 1 (Gas1) (**Figure 1A**). Hedgehog binding to its receptors relieves the repression exerted by Ptc on the G protein-coupled receptor Smoothened (Smo), which triggers either a complex intracellular signaling cascade involving the transcription factors of the Glioma-associated oncogene (Gli) family or mechanisms independent of Gli-mediated transcription known as ‘non-canonical’ (Ferent and Traiffort, 2015).

The present mini-review reports Hedgehog protein involvement in the development of oligodendroglial cells in the vertebrate spinal cord and brain from embryonic until neonatal stages. Moreover, the contribution of Hedgehog signaling to myelin repair will be discussed in the light of recent data implicating the transcription factor Gli1 and the receptor Smo.

## Hedgehog-Dependent OPC Production in the Spinal Cord

The involvement of Hedgehog morphogens in the specification of OPCs was proposed in the mid-1990s, when Sonic Hedgehog (Shh) signaling derived from the notochord and/or floorplate cells was found to be required for the development of the oligodendroglial lineage (Marti et al., 1995; Poncet et al., 1996; Pringle et al., 1996). Shh appeared to be both necessary and sufficient for expression of the genes encoding the oligodendrocyte transcription factors 1 (Olig1) and 2 (Olig2), associated with the oligodendroglial lineage in the motoneuron progenitor (pMN) domain of the developing neural tube. In the mouse, both genes are highly upregulated in the dorsal neural tube of embryonic day (E) 14.5 pups ectopically expressing Shh, just before the appearance of oligodendroglial cells (Lu et al., 2000). Consistent with this, in the zebrafish, several Smo mutants where most Hedgehog signaling is absent, exhibit an almost complete absence of oligodendrocytes in the spinal cord (Park et al., 2002; Schebesta and Serluca, 2009).

### Floorplate-Derived Hedgehog Proteins Contribute to OPC Specification

Although Shh is produced in both the notochord and floorplate, the rapid loss of contact between the notochord and the neural tube by E10.5–E11.5 led to analyze whether the floorplate could be the sole source of Hedgehog proteins during oligodendrogenesis. This single contribution was demonstrated by the selective inactivation of Shh in this area via mutagenesis approaches in the mouse. As soon as the E10.5 stage, the Olig2^+^ progenitors are significantly decreased in the mutant in a consistent manner with the requirement of Shh-derived floorplate to maintain the formation of this progenitor domain during neurogenesis. On later stages, while oligodendrogenesis occurs, several genes linked to gliogenesis including the fibroblast growth factor binding protein 3 (Fgfbp3) are also decreased as well as the Olig2 labeling that reaches a quite undetectable level at E12.5. The expression of OPC markers such as Nk2 homeobox 2 (Nkx2.2), Olig2, Sex determining region Y-box 10 (Sox10) and platelet-derived growth factor receptor alpha (Pdgfrα) is similarly reduced in the E15.5 mutant spinal cord (Yu et al., 2013). Unexpectedly, in the zebrafish, the syu^−/−^ mutant embryos, which are deficient for Shh, express Olig2 nevertheless more ventrally than normal and without giving rise to OPCs in the spinal cord. This species difference is related to the involvement of additional Hedgehog homologs (**Table 1**) including Indian Hedgehog b (Ihhb) and Tiggy winckle (Twhh) expressed in the notochord and/or floorplate during motoneuron and OPC specification (Krauss et al., 1993; Ekker et al., 1995; Park et al., 2002; Chung et al., 2013). Shh and Twhh both induce and position the Olig2^+^ precursor domain (Park et al., 2004) while Ihhb inhibits the cell cycle of the precursors implicated in OPC specification (Chung et al., 2013). Interestingly, in human, a transient high immunoreactive Shh signal is detected in the floorplate cells from 45 days embryos when the first OPCs emerge in two discrete close regions and progressively populate the whole spinal cord as observed in the other species (Hajihosseini et al., 1996).

**Table 1:** Hedgehog ligands in zebrafish, chick, rodent and human, and their activity related to the oligodendrocyte lineage.

Species	Hedgehog homologs	Activity in oligodendrogenesis	Reference
Zebrafish	Shha Shhb (Twhh) Ihha Ihhb (Echidna) Dhh	Induction and positionning of the Olig2^+^ precursor domain Induction and positionning of the Olig2^+^ precursor domain – Cell cycle inhibition of the precursors implicated in OPC specification –	Park et al., 2004 Chung et al., 2013
Chick	Shh Ihh Dhh	Induction of the oligodendroglial lineage – –	Marti et al., 1995; Poncet et al., 1996; Pringle et al., 1996
Rodent	Shh Ihh Dhh	Induction of the oligodendroglial lineage and Olig1/2 genes Potential redundant activity with Shh Potential redundant activity with Shh	Marti et al., 1995; Poncet et al., 1996; Pringle et al., 1996; Lu et al., 2000 Tekki-Kessaris et al., 2001
Human	Shh Ihh Dhh	Induction of OLIG2 and NKX2.2 in neural progenitors fated to the oligodendroglial lineage – –	Hajihosseini et al., 1996; Hu et al., 2009; Ortega et al., 2013

*The black dashes indicate the lack of any data regarding these homologs in oligodendrocyte development.*

### Motoneurons and OPCs Arise From Distinct Progenitor Cell Lineages in Response to Hedgehog

Nanomolar concentrations of Shh are able to induce with a similar efficiency both oligodendrocytes and motoneurons from neural tube explants (Pringle et al., 1996; Orentas et al., 1999; Lu et al., 2000). This observation was the starting point for a series of investigations aimed at determining if both cell types may be derived from a common precursor. However, the hypothesis was rapidly questioned. Data obtained from mouse and chick embryos indicated firstly that oligodendrocytes are generated at a later time than motoneurons, secondly that their precursors do not share the same transcription factors notably Nkx2.2 and the Paired box 6 protein (Pax6), respectively (Lu et al., 2000; Soula et al., 2001) and thirdly that Shh promotes the dorsal extension of the Nkx2.2 domain while it induces the regression of the Pax6 domain. More recent data carried out in zebrafish and using time-lapse imaging confirmed that the majority of motoneurons and oligodendrocytes are derived from distinct progenitors *in vivo*. In addition, they provided a unique mechanism involving the progressive recruitment of glial-fated progenitors to the pMN domain in a Hedgehog-dependent manner (Ravanelli and Appel, 2015). While progenitors located at the most ventral part of the pMN differentiate into motoneurons, more dorsal cells go on dividing. Moreover, progenitors located outside the pMN move ventrally and in turn upregulate Olig2 expression. Does such recruitment exist in mouse and chick is unknown. However, the recent finding that the pattern of the progenitor domains in the neural tube from those species depends on the regulation of the cell differentiation rates (Kicheva et al., 2014) is in support of this hypothesis. Moreover, the progenitor recruitment might account for a few intriguing observations including the maintenance of Olig2^+^ progenitors despite the loss of most motoneurons and OPCs in a mouse strain expressing a cell-lethal toxin in Olig1^+^ pMN progenitors (Wu et al., 2006).

### Molecular Mechanisms Driving the Hedgehog-Dependent Motoneuron/Oligodendrocyte Transition

The accumulation of Shh proteins at the surface of ventral neural progenitors occurred as a determining step in motoneuron/oligodendrocyte transition (Danesin et al., 2006). This accumulation was attributed to the activity of sulfatase1, which appears a short time before OPC specification in mouse and chicken embryos where the enzyme regulates the sulfation of heparin sulfate proteoglycans (HSPGs) (**Figure 1B**). By locally lowering Shh/HSPG interaction, sulfatase1 promotes Shh release from the progenitors located below the pMN domain providing higher Shh amounts to neighboring Nkx2.2^+^ cells before their transition to an oligodendroglial fate (Danesin et al., 2006; Touahri et al., 2012). Sulfatase2 coordinates its activity with sulfatase1 in this process (Jiang et al., 2017). In addition, fibroblast growth factors (Fgfs) initially thought to influence the Hedgehog-independent generation of dorsal oligodendrocytes occuring 3 days after the generation of pMN-derived OPCs, also participates in the creation of the burst of Shh required for ventral OPC specification (Farreny et al., 2018). In the zebrafish, sulfatase1 activity similarly stands as a timer activating neuronal and oligodendroglial generation at 14 and 36 h post-fertilization, respectively, in response to high concentrations of Hedgehog proteins (Al Oustah et al., 2014). Besides sulfatase enzymes, another mechanism essential for controlling the transition is the Shh-mediated restriction of the Notch ligand Jagged-2 (Jag2) to the pMN domain during motoneuron generation. Indeed, Jag2 expression prevents a subset of Olig2^+^ progenitors from differentiating into neurons and maintains a high expression of bHLH transcription factor 5 (Hes5), one of the Notch effectors that inhibits the generation of OPCs during the neurogenic step (Rabadan et al., 2012).

### Hedgehog Signaling Components Implicated in OPC Specification

The involvement of the positive and negative effectors of Shh signaling, the Gli zinc-finger transcription factors Gli2 and Gli3, was addressed in the induction of OPCs mediated by Shh. The analysis of the mutant mice revealed that Gli2 is implicated in the regulation of the size and duration of the Olig1/2^+^ domain in the ventral neuroepithelium, but not in the proliferation, differentiation and maturation of OPCs after their initial production (Qi et al., 2003). In contrast, a non-essential role was attributed to Gli3 in ventral oligodendrogenesis in agreement with the restriction of Gli3 expression to the dorsal spinal cord at E9.5 (Lee et al., 1997) and thus before oligodendrogenesis starts. Interestingly, the Gli3 null mutation was nevertheless found to substantially rescue the phenotype exhibited by mice deprived of Shh in the floorplate (Yu et al., 2013) suggesting that Shh likely maintains Olig2 expression in OPCs by repressing the antagonistic Gli3 repressor activity. On the contrary, the regulation of Nkx2.2 expression was proposed to likely rely on the activation of Gli activator forms (Oh et al., 2005; Tan et al., 2006; Yu et al., 2013).

## Hedgehog-Dependent OPC Production in the Brain

The origin of oligodendrocytes in the developing brain has long remained less well-established than in the spinal cord. Hedgehog signaling implication in OPC generation was progressively investigated at the different rostro-caudal levels and finally detected not only in the ventral, but also in the dorsal brain.

### Hedgehog-Dependent OPC Generation in the Hindbrain and Midbrain

The first data were reported in the chick hindbrain where ventral and dorsal domains from which OPCs arise are correlated with a transient Shh expression (Ono et al., 1997; Davies and Miller, 2001). In the rodent hindbrain, like in the spinal cord, ventral OPCs depend on Shh signaling and require Olig1/2 expression (Lu et al., 2000; Alberta et al., 2001; Vallstedt et al., 2005). However, a crucial difference relies on the opposing effects mediated by the Nkx6 proteins, which are required for OPC generation in the spinal cord but on the contrary suppress the production of OPCs in the anterior hindbrain (Vallstedt et al., 2005). In the zebrafish, the homozygous Smo mutant completely lacks hindbrain oligodendrocytes (Park et al., 2002). In this species, both Olig2 and Nkx2.2a expression depend on Hedgehog signaling via a mechanism implicating histone deacetylase 1 proposed to facilitate Hedgehog-mediated expression of Olig2 and to repress Nkx2.2a in neural progenitors fated to the oligodendrocyte lineage (Cunliffe and Casaccia-Bonnefil, 2006). Moreover, the unexpected identification of Disrupted-in-schizophrenia1 as a target of Shh signaling during OPC specification, interestingly suggested that impaired Shh signaling may constitute one of the potential developmental factors involved in the pathobiology of mental illnesses (Boyd et al., 2015).

In the midbrain, much less data are available. The development of ventral and dorsal OPCs mainly examined in chicken was proposed to depend on Shh (Fu et al., 2003). A unique feature is that dorsal Olig2^+^ OPCs start to co-express Nkx2.2 only when they migrate away from the VZ. Moreover, in the cerebellum, most OPCs have been proposed to arise from a small region called the parabasal bands (Mecklenburg et al., 2011). Although Shh implication in OPC specification in this area is not known, Purkinje cell-derived Shh in rodent was found to prevent OPCs from exiting the cell cycle and to inhibit the effects of molecules inducing their differentiation (Bouslama-Oueghlani et al., 2012).

### Hedgehog-Dependent OPC Generation in the Ventral Forebrain

In the mouse forebrain, three successive waves of OPCs were reported. They arise at E12.5, E15.5, and P0 from Nkx2.1, Genetic-screened homeobox 2 (Gsh2) and homeobox protein Emx1 progenitors respectively located in the medial ganglion eminence, lateral ganglion eminence and cerebral cortex (Kessaris et al., 2006) (**Figure 1C**). In contrast to the spinal cord, most OPCs present in the adult forebrain, are dorsally-derived (Tripathi et al., 2011).

Shh signaling was early proposed to be central to the generation of ventral forebrain OPCs. In a consistent manner, the lack of the telencephalic Shh expression domain in the Nkx2.1 null mutant mice is associated with an absence of OPCs in the ventral forebrain, a phenotype rescued by *in vivo* Shh gain-of-function experiments. However, although Shh is able to direct cells toward an oligodendroglial fate, additional local signals appeared to be required for their differentiation into mature oligodendrocytes (Alberta et al., 2001; Nery et al., 2001; Tekki-Kessaris et al., 2001; Qi et al., 2002). Consistent data were obtained in the zebrafish since the syu^−/−^ mutant displays a lower level of Olig2 expression (Park et al., 2002).

### Hedgehog-Dependent OPC Generation in the Dorsal Forebrain

Shh-dependence of dorsal forebrain OPCs was in contrast only recently demonstrated notably by the report of a Shh-dependent domain in the dorsal V-SVZ producing large numbers of oligodendrocytes at the neonatal period. Indeed, an adenoviral lineage tracing showed that dorsal V-SVZ-derived neural progenitors expressing Gli1 in neonates differentiate into mature oligodendrocytes (Tong et al., 2015) and give rise to myelinating-like membranes (Sanchez and Armstrong, 2018). Moreover, the adenovirus-mediated ablation of Smo or its ectopic activation in those progenitors reduces or conversely increases the generation of mature oligodendrocytes in the corpus callosum (Tong et al., 2015). These data are fully consistent with previous *in vitro* results obtained by using neocortical NSC cultures derived from late embryo stages. Cell treatment with a Smo agonist induces an increase in Olig2^+^ cells that can be blocked by the well-known Smo antagonist cyclopamine (Kessaris et al., 2004). Furthermore, Gli1 (but not Gli2 or Gli3) is upregulated when exogenous Shh is added to the culture (Wang et al., 2008). However, all these data apparently disagree with the precocious myelination observed in 9-day old Gli1 null mutant mice unexpectedly suggesting that Gli1 delays the onset of myelination (Samanta et al., 2015). One of the main differences between these apparently discordant experiments is a full invalidation of Gli1 as soon as the embryonic period on one side and, a conditional and postnatally induced removal of Smo in the dorsal NSCs on the other side. For this reason, the phenotype of the Gli1 mutant could reflect the activity of Gli1 before birth, which might consist in maintaining OPCs in the cell cycle thus preventing their differentiation into myelinating oligodendrocytes. However, if this hypothesis is true, Gli1 activity before birth should be independent on Smo given the compromised generation of oligodendrocytes observed upon conditional removal of Smo in nestin-expressing embryonic NSCs (Machold et al., 2003). For instance, the capacity of Gli1 to antagonize Gli3 previously shown in the spinal cord (Bai and Joyner, 2001) might be a mechanism interesting to be investigated in the forebrain. Beside this first activity of Gli1, a second one could exist at a lower extent in embryo and could be maintained at the perinatal period when most myelinating oligodendrocytes are generated. Instead preventing myelination, Gli1 might then participate in the proliferation/differentiation of NSC and/or OPCs. In agreement with this hypothesis, a dual and context-dependent function has been proposed for Gli1 in the maintenance of NSC proliferation during conditions of self-renewal and in their maturation into oligodendroglial cells during differentiation (Wang et al., 2008). The phenotype observed upon conditional removal of Smo at birth is consistent with this second activity of Gli1 that would then depend on Smo. How these Gli1 activities might be triggered remains to be determined. The involvement of other Hedgehog proteins (Dhh or Ihh) cannot be excluded if we consider that the generation of oligodendroglial cells is maintained in Shh null mutant mice (Nery et al., 2001) and that embryonic cortical NSC cultures upregulate Shh, Ihh and at a lower extent Dhh (Tekki-Kessaris et al., 2001). However, an accurate analysis of each step of dorsal oligodendrogenesis in the absence of Gli1 or Smo separately or simultaneously would of course be required to evaluate all these hypotheses.

### Molecular Mechanisms Implicated in Hedgehog-Dependent Production of OPCs in the Forebrain

From a molecular point of view, Shh-dependent oligodendrogenesis in the spinal cord and forebrain display both similarities and divergences. In contrast to the spinal cord, the control of OPC production in the forebrain is Gli2-independent even though the role of Gli2 in the terminal differentiation of oligodendrocytes remains unclear (Qi et al., 2003). Conversely, in both regions, Shh and BMP display antagonistic activities on OPC production. In cultured OPCs derived from the forebrain, Shh and Bone morphogenetic protein 4 (BMP4) favor and inhibit respectively the progression toward oligodendrocytes. While the former decreases histone acetylation and induces peripheral chromatin condensation, the latter promotes global histone acetylation and retains euchromatin thus favoring astrogliogenesis (Wu et al., 2012). Moreover, Shh-mediated OPC production in the forebrain is tightly associated with Fgf as previously shown in the spinal cord. Consistent with this, precursors isolated from E14 mouse medial ganglion eminence, responding to Platelet-derived growth factor (Pdgf) and giving rise to both neurons and OPCs are able to self-renew via Pdgf and Fgf2-mediated activation of Shh signaling (Chojnacki and Weiss, 2004). Similarly, a basal level of phosphorylated mitogen-activated protein kinases (MAPKs) maintained by Fgf activity is required for Shh-induced generation of OPCs from cultures of E13.5 mouse neocortical precursors (Kessaris et al., 2004; Furusho et al., 2011). The absence of a significant effect in Shh signaling in the Fgf receptors knockout mice as well as the strong inhibition of OPC generation in cell cultures from E11.5 ventral forebrains by inhibitors of Fgf and Shh signaling used individually or in combination led to conclude to the cooperation of those signaling pathways for the generation of most ventrally derived OPCs (Furusho et al., 2011). A comparable crosstalk between Shh and Fgf signaling also exists in the zebrafish where Fgf16 required for oligodendrogenesis in the forebrain, is induced by Hedgehog signaling (Miyake et al., 2014). Interestingly, in human, OPCs originate both from the ventral telencephalon and the cortical SVZ at midgestation (Rakic and Zecevic, 2003; Jakovcevski et al., 2009). Human fetal neural progenitors derived from the cortical SVZ give rise to oligodendrocytes *in vitro* in a Shh-dependent manner (Jakovcevski et al., 2009; Mo and Zecevic, 2009; Ortega et al., 2013). However, the transition of pre-OPCs to OPCs is inhibited by FGF2, which represses SHH-dependent expression of OLIG2 and NKX2.2 (Hu et al., 2009).

### Hedgehog-Dependent OPC Generation in the Optic Nerve

Oligodendrocyte progenitor cells present in the optic nerve are derived from cells located in the floor of the third ventricle (Small et al., 1987; Ono et al., 1997). The first cells are generated concomitantly with the arrival of retinal axons to the forebrain under the activity of Shh transported in the chick from the retinal ganglion cells to the optic chiasm via the retinal axons (Gao and Miller, 2006). Cultures of mouse optic nerve explants and Shh-interference *in ovo* in chick embryo showed that Shh also acts as a mitogen and a chemoattractant for optic nerve OPCs (Merchan et al., 2007). Since Shh is synthesized in the floor of the third ventricle, how newly generated OPCs are not retained in this area remains however an open question. Moreover, an interesting mechanism proposed for controling the level of Shh available for OPCs during the colonization of the optic nerve, relies on the internalization of Shh via the multiligand receptor Megalin before Shh release by astrocytes (Ortega et al., 2012).

## Hedgehog Signaling in Myelin Regeneration

Given that the first step of myelin regeneration following a demyelinating insult involves the reactivation of quiescent OPCs as well as the re-expression of genes essential during development (Franklin and Ffrench-Constant, 2008), the contribution of Shh signaling to OPC production in the context of CNS demyelination has been addressed by several groups. After the report that exogenous Shh is able to increase OPCs and premyelinating oligodendrocytes in the adult healthy dorsal forebrain (Loulier et al., 2006), various demyelination models led to show Shh signaling implication in myelin repair. The administration of interferon-β was found to promote the upregulation of Shh and its receptor Ptc likely leading to the inhibition of the negative effects of Notch signaling in models of immune and non-immune demyelination (Mastronardi et al., 2004). Along the same line, the capacity of thyroid hormones to improve clinical signs and remyelination upon chronic demyelination was proposed to be related to a potent OPC induction probably due to the increase of Shh expression (Harsan et al., 2008). Consistent with all these data, gain- and loss- of function experiments led to demonstrate in the lysolecithin-mediated demyelination of the corpus callosum, that Shh promotes proliferation and differentiation of OPCs and decreases astrogliosis and macrophage infiltration altogether leading to the attenuation of the lesion extent during myelin repair (Ferent et al., 2013). Although Shh upregulation is a shared feature in all these works, genetic fate-labeling of cells actively transcribing Shh failed to detect Shh-expressing cells following cuprizone-mediated demyelination (Sanchez et al., 2018). One hypothesis able to account for this discordance could be that the antibodies used for visualizing Shh have actually recognized other members of the Hedgehog family (Traiffort et al., 2001). The hypothesis remains to be investigated.

More recently, the involvement of Smo and Gli1 was also addressed in the context of myelin regeneration. In agreement with the pro-regenerative effects previously proposed for the Shh pathway (see above), the activation of Smo by micro-injection of a Smo agonist into the corpus callosum of mice chronically demyelinated by cuprizone administration increases cell proliferation and enhances remyelination. However, no significant increase in cycling OPCs or oligodendrocytes was reported. Moreover, the absence of Gli1 fate-labeled cell increase in this experimental paradigm led to suggest that the Smo agonist promotes remyelination independently of Gli1 possibly indicating signaling through the non-canonical Hedgehog pathway and, likely in indirect manner by acting on cells other than the oligodendroglial cells (Sanchez et al., 2018). Conversely, Vismodegib, a specific Smo antagonist, reported to repress Gli-mediated transcription in a variety of cell types (Stanton and Peng, 2010) significantly increases disease severity in the experimental autoimmune encephalomyelitis (EAE) model of demyelination. However, the data did not evaluate the state of Gli1 activation in these animals (Alvarez et al., 2011).

Whereas the modulation of Smo activity is consistent with the positive effect of Shh signaling on remyelination, more controversial data emerged from the investigation of the roles of Gli1. Indeed, the genetic fate-labeling of cells expressing Gli1 led to detect a subset of Shh-responsive adult NSCs comprising 25% of all NSCs present in the ventral V-SVZ. Upon cuprizone administration, these cells can be recruited to the demyelinated corpus callosum where they are preferentially fated to the oligodendrocyte lineage and concomitantly downregulate Gli1 expression (Samanta et al., 2015; Sanchez et al., 2018). Moreover, the genetic downregulation of Gli1 or its pharmacological inhibition in the cuprizone model was found to amplify the recruitement of NSCs, increase their differentiation into mature oligodendrocytes and enhance the density of myelin basic protein signal in the demyelinated CNS. Similarly, the pharmacological inhibition of Gli improves the functional outcomes in the EAE model by promoting remyelination and neuroprotection in the spinal cord through direct or indirect effects on Gli1-expressing NSCs or parenchymal astrocytes, respectively (Samanta et al., 2015). If the remyelinating effects induced by Smo activation are mediated via the non-canonical (Gli-independent) pathway as previously suggested (Sanchez et al., 2018), the pro-regenerative effects observed in the presence of a Gli antagonist (Samanta et al., 2015) do not constitute discordant results. However, it is noteworthy that, during the demyelination/remyelination processes, Gli1 expression appears to be regulated in a complex manner depending on the animal model or the stage of the disease. Thus, in the cuprizone model, Gli1 was reported to be not or slightly upregulated in the demyelinated corpus callosum mainly in reactive astrocytes (Samanta et al., 2015; Sanchez et al., 2018). In the lysolecithin-induced demyelination of the corpus callosum, a quite moderate Gli1 transcription is observed in oligodendroglial cells during the step of OPC differentiation (Ferent et al., 2013). In the EAE model, Gli1 is upregulated in OPCs and neurons just before EAE onset and then downregulated during the irreversible demyelination occurring in this model (Wang et al., 2008). Interestingly, the kinetics of Gli1 transcription determined in the EAE model reminds the regulation of Gli1 expression determined in the brain from multiple sclerosis patients as shown by the upregulation of Gli1 in active lesions and its decrease in chronic active and inactive lesions (Wang et al., 2008). Remarkably, the expression of Gli1 in tissues able to remyelinate is in support of a potential pro-myelinating role of Gli1. Besides its negative role on remyelination and neuroprotection targeting a subset of NSCs, the pro-regenerative role of Gli1 would thus deserve to be investigated. According to the demyelination model, the mouse strain, the methodological approaches leading to Gli1^+^ cell tracking, or the analysis of tissues derived from the human disease, the phenotype of the cells expressing Gli1 or Smo is variable including NSCs, reactive astrocytes, oligodendroglial cells or even neurons for Gli1, microglia, oligodendroglial cells, and astrocytes for Smo. Therefore, the apparently contradictory results showing that the blockade (Gli inhibition) and the activation (Shh overexpression or Smo activation) of Shh signaling both finally improve remyelination may reflect not only the potential involvement of canonical and non-canonical Shh signaling pathways, but also the targeting of different remyelinating cells (NSCs and parenchymal OPCs) and probably the differential regulation of reactive astrocytes and activated microglia. The observation that the blockade of Gli1 in the NSCs preferentially fated to oligodendroglia appears to be endowed with an even greater effect in the context of active Shh signaling (Samanta et al., 2015) undoubtedly deserves further investigations.

## Conclusion

The data accumulated over the years on the role of Hedgehog signaling in the genesis of the oligodendrocyte lineage clearly demonstrates that Hedgehog stands as one of the important pathways in this process. However, many questions remain regarding the apparent intricacy of Hedgehog signaling activity during myelin regeneration. The thorough determination of the molecular mechanisms and cell types targeted by the pharmacological modulation of the different components of the pathway should improve our understanding and ultimately open new therapeutic perspectives.

## Author Contributions

ET and YL contributed to the design, documentation, and writing of this review article.

## Conflict of Interest Statement

The authors declare that the research was conducted in the absence of any commercial or financial relationships that could be construed as a potential conflict of interest.
